# Uveal melanoma with extrascleral extension and systemic metastasis complicating into retinal detachment and cataract formation

**DOI:** 10.4103/0301-4738.62663

**Published:** 2010

**Authors:** Shivcharan L Chandravanshi, Sujata Lakhtakia, Mahesh K Rathore, Prem C Dwivedi, Jainendra S Rahud

**Affiliations:** Department of Ophthalmology, Gandhi Memorial Hospital, Rewa 486001 M.P., India

Dear Editor,

The article by Khetan *et al.* on an unusual presentation of uveal melanoma was interesting and the authors deserve congratulations for reporting it. However, we wish to make the following observations on this rare case report.

1. Authors have mentioned that magnetic resonance imaging (MRI) confirms the diagnosis of choroidal melanoma. At many times MRI is confusing in the diagnosis of choroidal lesions and gives only radiological clues whereas histopathological examination confirms the diagnosis.[2]
2. “Presenting as” is a common phrase in medical writing used to describe any unusual presentation of disease. Authors have highlighted uveal melanoma presenting as cataract and staphyloma. Staphyloma is a localized bulging of weak and thin outer tunic of the eyeball, lined by uveal tissue which shines through it. In their case, there is “almost a total loss of scleral fibers in the entire nasal quadrant” and tumor mass is visible through the scleral defect. Therefore it doesn't fit into the definition of staphyloma because there is neither scleral ectasia nor normal uveal tissue. Hence it is more likely a case of uveal melanoma with extrascleral extension. The authors have described anterior segment examination of right eye to be within normal limits but they have not mentioned its intraocular pressure (IOP). This is important since fundus findings of the same eye are suggestive of primary open angle glaucoma (POAG) and IOP of the left eye was 36 mm of Hg.
3. Cataract formation can occur in uveal melanoma due to lenticular infiltration by tumor cells which can be confirmed by histological examination of the lens.[3] Patient has fundus findings suggestive of POAG in the right eye and therefore there is a possibility of pre-existing bilateral POAG which could be another probable mechanism of cataract formation. The third possible mechanism may be secondary angle closure glaucoma secondary to neovascularization of iris and angle of anterior chamber.
4. Authors have speculated the origin of uveal melanoma in a pre-existing blind eye. But considering the six months duration of blindness and the slow-growing nature of malignant melanoma, it appears to be the reverse i.e. uveal melanoma leading to blindness.[4] Thus, advanced malignant melanoma leading to retinal detachment and cataract formation is more likely the cause of blindness in this case rather than origin of uveal melanoma in a blind eye.
5. Authors have mentioned “modified extended enucleation” under general anesthesia but they have neither described nor referenced the technique. An exhaustive English language PubMed search did not show any publication on “modified extended enucleation” technique. However, there are many modifications of enucleation. The “No-touch” technique modification is advocated by Fraunfelder and Wilson for enucleation of eyes that harbor intraocular malignancy.[5] Higher IOP secondary to manipulation of eyeball can lead to dissemination of tumor cells into the bloodstream and widespread metastasis. However, in the “no-touch technique, no direct pressure is exerted on the eyeball during enucleation and the tumor is frozen with intermittent cryo treatment to prevent metastatic spread.
